# Phenotypical Differentiation of Tremor Using Time Series Feature Extraction and Machine Learning

**DOI:** 10.1002/mds.70032

**Published:** 2025-09-05

**Authors:** Verena Häring, Veronika Selzam, Juan Francisco Martin‐Rodriguez, Petra Schwingenschuh, Gertrúd Tamás, Linda Köhler, Jan Raethjen, Steffen Paschen, Franziska Goltz, Eoin Mulroy, Anna Latorre, Pablo Mir, Rick C. Helmich, Kailash P. Bhatia, Jens Volkmann, Robert Peach, Sebastian R. Schreglmann

**Affiliations:** ^1^ Department of Neurology University Hospital Würzburg Würzburg Germany; ^2^ Unidad de Trastornos del Movimiento, Servicio de Neurologia y Neurofisiologia Clinica, Instituto de Biomedicina de Sevilla Hospital Universitario Virgen del Rocio/CSIC/Universidad de Sevilla Seville Spain; ^3^ Centro de Investigación Biomédica en Red sobre Enfermedades Neurodegenerativas (CIBERNED), Instituto de Salud Carlos III Madrid Spain; ^4^ Departamento de Psicología Experimental, Facultad de Psicología Universidad de Sevilla Seville Spain; ^5^ Department of Neurology Medical University of Graz Graz Austria; ^6^ Department of Neurology Semmelweis University Budapest Hungary; ^7^ Department of Neurology University Hospital Schleswig‐Holstein, Campus Kiel, Christian Albrechts University Kiel Germany; ^8^ Department of Neurology, Donders Institute for Brain, Cognition and Behaviour Radboud University Medical Center Nijmegen The Netherlands; ^9^ Department of Clinical and Movement Neurosciences UCL Queen Square Institute of Neurology London UK; ^10^ Departamento de Medicina, Facultad de Medicina Universidad de Sevilla Seville Spain; ^11^ Department of Brain Sciences Imperial College London London UK

**Keywords:** tremor analysis, essential tremor, Parkinson's disease, big data

## Abstract

**Background:**

The clinical diagnosis of tremor disorders depends on the interpretation of subtle movement characteristics, signs, and symptoms. Given the absence of a universally accepted biomarker, differentiation between essential tremor (ET) and tremor‐dominant Parkinson's disease (PD) frequently proves to be non‐trivial.

**Objective:**

To identify generalizable tremor characteristics to differentiate ET and PD using feature extraction and machine learning (ML).

**Methods:**

Hand accelerometer recordings from 414 patients, clinically diagnosed at six academic centers, formed an exploratory (158 ET, 172 PD) and a validation dataset (30 ET, 54 PD). Established, standardized tremor characteristics were assessed for their cross‐center accuracy and validity. Supervised ML was applied to massive higher‐order feature extraction of the same recordings to achieve optimal stratification and mechanistic exploration.

**Results:**

While classic tremor characteristics did not consistently differentiate between conditions across centers, the feature combination identified via our ML approach was successfully validated. In comparison with the tremor stability index (TSI), feature‐based analysis provided better classification accuracy (81.8% vs. 70.4%), sensitivity (86.4% vs. 70.8%), and specificity (76.6% vs. 70.2%), substantially improving disease stratification. The interpretation of identified features indicates fundamentally different dynamics in tremor‐generating circuits: while different discrete but stable signal states in PD indicate several central oscillators, signal characteristics in ET point towards a singular pacemaker.

**Conclusion:**

This study establishes the use of feature‐based ML as a powerful method to explore accelerometry‐derived tremor signals. The combination of hypothesis‐free, data‐driven analyses and a large, multicenter dataset represents a relevant step towards big data analysis in tremor disorders. © 2025 The Author(s). *Movement Disorders* published by Wiley Periodicals LLC on behalf of International Parkinson and Movement Disorder Society.

Tremor, the most frequent movement disorder,^1^ predominantly affects the hands, causing considerable impairment.^2^ Essential tremor (ET) and Parkinson's disease (PD) are the most common etiologies. ET is a relatively static condition, largely believed to originate in the cerebellum,^3^, ^4^, ^5^ and is characterized by an isolated, bilateral postural or kinetic hand tremor,^6^ but sometimes also rest tremor.^7^, ^8^ Conversely, PD is a progressive neurodegenerative disease characterized by bradykinesia, rigidity, and a tremor that typically occurs during rest, but often also re‐emerges during posture.^9^, ^10^, ^11^, ^12^ The pathophysiology of PD tremor is thought to involve both the basal ganglia and the cerebello‐thalamo‐cortical circuit.^13^, ^14^

Historically, descriptive studies established classification systems based on classic tremor phenotypes,^15^, ^16^, ^17^, ^18^, ^19^ but significant phenotypical overlap^20^ causes misdiagnoses and mistreatment.^21^, ^22^ Therefore, an increasing number of studies employ inertial measurement units^23^, ^24^ for tremor evaluation^23^.^25^, ^26^, ^27^

To date, two validated methods differentiate ET from PD tremor: (1) The tremor stability index (TSI), a metric of the relative range of frequency tolerance of a tremor, was established to compare PD *rest* tremor versus ET *postural* tremor recordings, offering best differentiation at a TSI cut‐off of 1.05.^28^ However, absolute TSI values have been found to differ in other cohorts,^29^, ^30^ casting doubt on its generalizability. (2) The mean harmonic power,^31^ a metric quantifying the power at higher harmonic frequencies of the main tremor frequency, was established based on postural recordings and validated in three independent cohorts.^28^, ^32^ Still, these metrics seem unable to consistently differentiate tremor disorders.^25^, ^33^ Meanwhile, the widespread use of accelerometers in studies^34^ and clinical routine^26^ allow the aggregation of large, multicenter datasets to search for unifying, generalizable signal characteristics to differentiate tremor disorders.

Machine learning (ML) describes the process of identifying patterns from data without human interference. This analytical concept is transforming medical research,^35^, ^36^, ^37^ integrating complex datasets by automatic pattern recognition.^29^, ^30^, ^31^ ML is already applied to tremor research,^33^ but only sparsely to etiological stratification.^38^, ^39^, ^40^, ^41^, ^42^ Moreover, issues such as limited sample sizes, monocentric study designs, and the constraints of hypothesis‐driven approaches have hindered the identification of reliable, generalizable disease‐specific tremor characteristics.^33^

Our study identifies generalizable tremor characteristics that differentiate PD and ET patients by combining a large multicenter dataset with a hypothesis‐free approach. By combining massive feature extraction^43^ with ML,^44^ we identified previously unrecognized disease‐ and state‐specific movement characteristics. Altogether, this method is advancing the accurate classification of PD and ET and represents a novel tool for unbiased time series analysis.

## Methods

### Participants and Ethical Approval

Accelerometry recordings from patients with clinically established diagnoses of PD (n = 226) or ET (*n* = 188) collected at six centers with movement disorder expertise (Graz, Budapest, London, Kiel, Nijmegen, and Wuerzburg),^31^, ^45^, ^46^ were included. Clinical and demographic details were available from all centers, apart from the historical cohort from Kiel (Table S1). All patients were diagnosed based on at least two independent clinical consultations according to the Consensus Statement on Classification of Tremor,^17^, ^18^ apart from the Nijmegen PD patients, who were diagnosed on a singular visit.^47^ All participants provided written informed consent according to local ethics at each participating center prior to inclusion. The overall analysis was approved by the local research ethics committee (IRB University of Würzburg, Nr. 20210209 03) in accordance with the Declaration of Helsinki.

### Accelerometer Examination

Across sites, accelerometer recordings were performed after an overnight withdrawal of tremor‐influencing medication. Bilateral rest and posture recordings (triaxial in Graz, Budapest, London, Nijmegen, Wuerzburg; monoaxial in Kiel) were performed according to local operating procedures^28^, ^45^, ^46^ with center‐specific slight variations in recording positions and equipment (Table S2). Recordings exhibiting a change in tremor frequency > 1 Hz upon loading with 500 g/1000 g were removed to exclude enhanced physiological tremor.^27^, ^48^

### Data Analysis

#### Raw Accelerometer Data Preparation

**FIG. 1:**
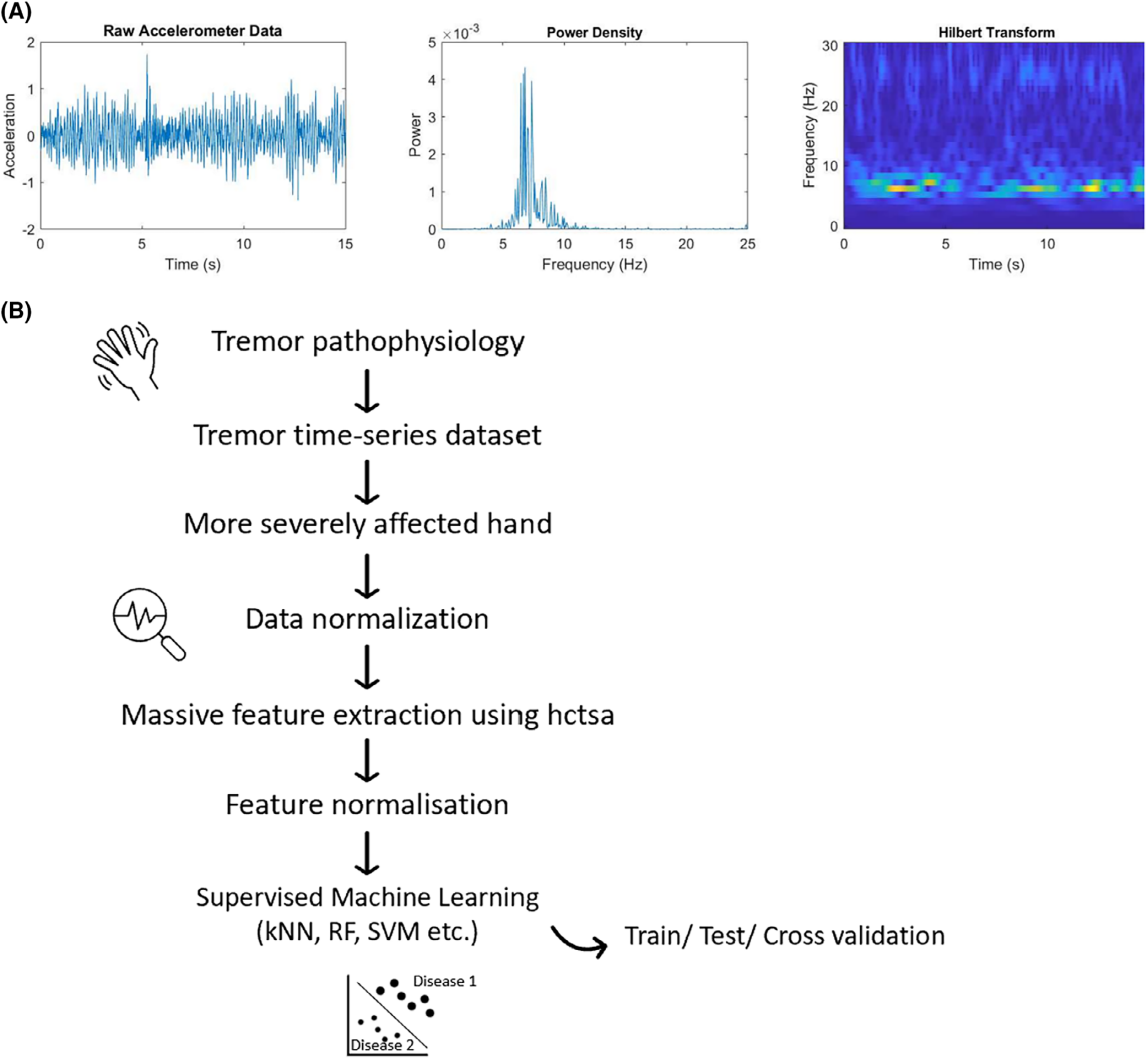
Analytical pipeline from tremulous movement and raw accelerometry signal to supervised statistical learning. (A) Tremor time series data include a wealth of information in their raw format, which can be conceptualized in frequency (power density) and time domain (Hilbert transform/wavelet). (B) Scheme illustrating the steps from recording tremulous hand movements via an inertial measurement unit, extraction of higher mathematical features, and machine learning to validation. [Color figure can be viewed at wileyonlinelibrary.com]

All analyses were performed in Matlab (R2021b; Mathworks, Natick, MA, USA). All time series raw data and their conversion in frequency and time domain (Fig. 1A) were individually visualized and screened for artefacts. For triaxial accelerometer data the vector amplitude sum was used for analyses, while monoaxial recordings were used as they were.

(1) $$A=\sqrt{x^{2}+y^{2}+z^{2}}$$

All data were normalized to a mean of 0 and standard deviation of 1, down sampled to 100 Hz, standardized to a recording length of 15 s, and bandpass filtered between 2 Hz and 30 Hz.

We consistently used recordings from the more affected hand in order to (1) circumnavigate the caveat of potentially just measuring tremor asymmetry, (2) obtain more mechanical information, and (3) remove potential spurious influences of picking random hands by calculating the relative side differences of the area under the curve (AUC) – a proxy for tremor power – in both rest and postural positions. The position with the largest relative AUC difference between sides was then used as the more affected hand:

(2) $${\mathrm{AUC}}_{\mathrm{rel}}=\frac{{\mathrm{AUC}}_{\max\_\text{right}}-{\mathrm{AUC}}_{\max\_\text{left}}}{{\mathrm{AUC}}_{\max\_\text{right}}+{\mathrm{AUC}}_{\max\_\text{left}}}$$

#### Standard Tremor Characteristics

Frequency‐domain‐based standard tremor characteristics were calculated as previously described^28^, ^30^: area under the curve (AUC), tremor stability index (TSI), half width power (HWP), peak frequency, full‐width half maximum (FWHM), and peak power.

#### Automated Feature Extraction and ML

To increase the analytical scope, we applied an automatic massive feature extraction approach not limited by predetermined characteristics but explored the entirety of mathematical descriptors of the time series data^44^ (Fig. 1B). In short, we used the highly comparative time series analysis (hctsa) algorithm^43^, ^49^ to compute a large number of features including autocorrelations, power spectra, wavelet decompositions, distributions, time series models, information‐theoretic quantities, and nonlinear measures for each time series (full documentation of features: https://time-series-features.gitbook.io/hctsa-manual/.) After removing features with infinity, not a number (NaN) values or zero variance, this resulted in a feature count of 7729. To remove the impact of variance in feature scale, the value of each feature was individually normalized to the interval [0,1]. The resulting features were then used as high‐dimensional input for ML algorithms.

#### Model Evaluation

To compare the classification accuracy of different supervised ML algorithms, data were split into train and test partitions and the balanced classification accuracy computed for each algorithm:

(3) $${\text{Accuracy}}_{\text{classification}}=\frac{\text{number of accurate classifications}}{\text{number of}\;\mathrm{all}\;\text{classifications}}$$

This was validated by a 10‐fold cross‐validation, ensuring that recordings from each patient were in the same fold. The most relevant features were identified via a univariate approach and selected for further analysis. After detecting relevant differences between monoaxial and triaxial recordings, we optimized the classification process and applied standard and ML‐based comparisons to all triaxial recordings from an explorative dataset (Graz, Budapest, London) and two separate validation datasets (Nijmegen, Wuerzburg), referred to as *final data*.

We next identified features with >75% univariate classification accuracy and assessed all possible combinations of two, three, and four features for their combined classification accuracy in normalized as well as non‐normalized time series data to find features dependent as well as independent of tremor amplitude.

#### Statistical Analysis

Statistical testing included the Kolmogorov–Smirnov test, Fisher's exact test, and Wilcoxon rank‐sum test in MatLab, as well as one‐way analysis of variance (ANOVA) in JASP (JASP Team 2023). All tests were two‐tailed, and the α level was set at *P* < 0.05. All *P‐*values were Bonferroni‐corrected.

## Results

### Patient Cohorts

In this study, we analyzed data and accelerometer recordings from 414 tremor patients, with detailed demographic information (Graz [17 ET, 21 PD],^46^ Budapest [36 ET, 47 PD], London [6 ET, 5 PD], Nijmegen [9 ET, 31 PD),^50^ Wuerzburg [21 ET, 23 PD], and a historical sample from Kiel [99 ET, 99 PD]; Table S1).

Among the 216 patients for whom demographic information was available, ET and PD patients were matched for age and sex. The onset of tremor occurred at a younger age in ET (37.99 ± 23.29 years) compared with PD patients (57.02 ± 11.79 years; *P* = 0.1*10^−5^) and the mean disease duration was longer in ET (24.13 ± 20.44 years) than PD patients (6.54 ± 6.45 years; *P* = 1.1*10^−11^).

### Comparison of Standard Tremor Characteristics

**FIG. 2:**
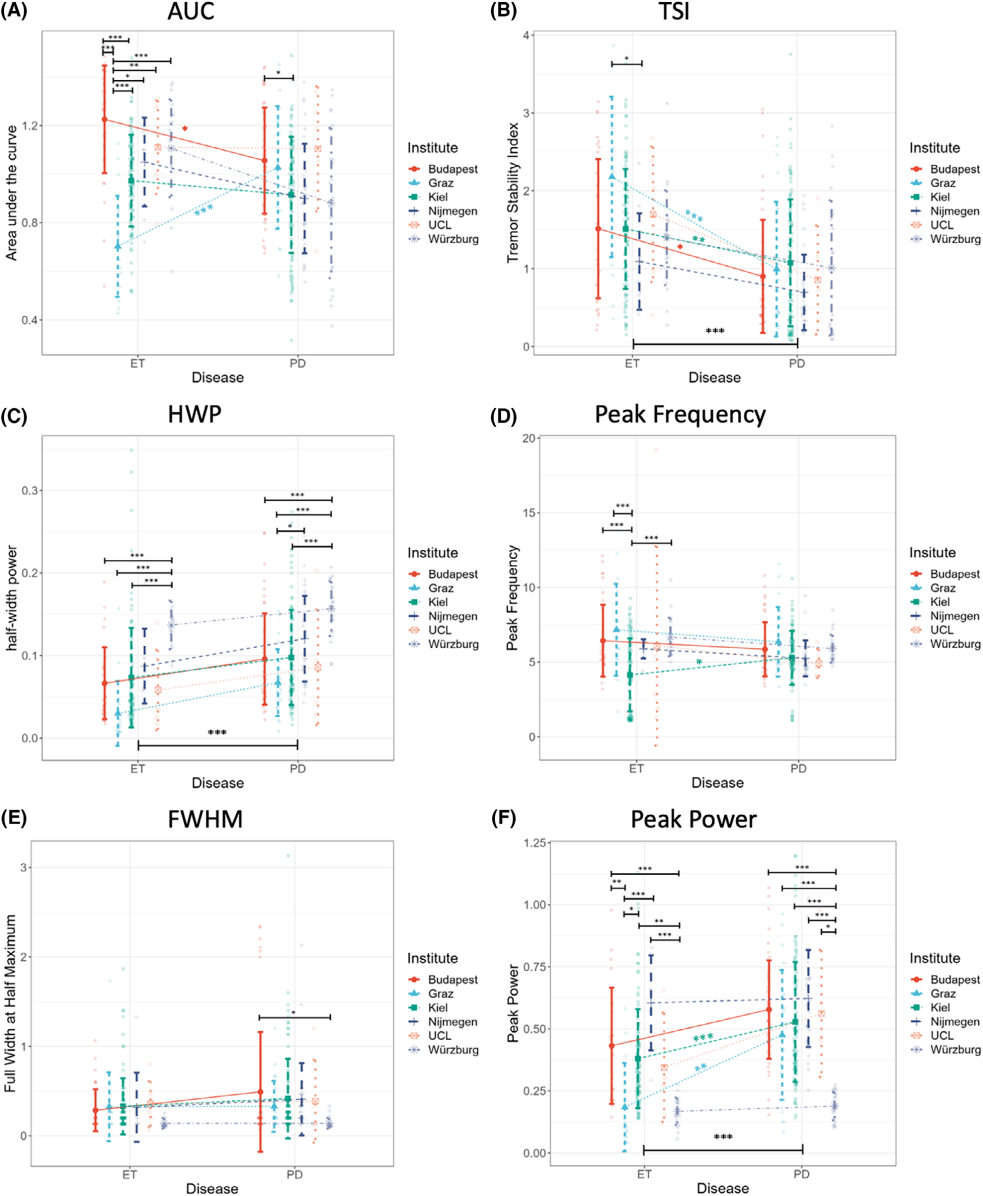
Consistency and generalizability of standard tremor characteristics in their ability to differentiate essential tremor (ET) and Parkinson's disease (PD) tremor between and across centers. For the characterization of patient cohorts, established tremor characteristics including (A) area under the curve (AUC), (B) tremor stability index (TSI), (C) half‐width power (HWP), (D) peak frequency, (E) full‐width half maximum (FWHM), and (F) peak power were calculated and compared between centers and diseases (results from rest recordings shown). The comparison of HWP, peak power, and TSI resulted in group differences between disorders. Notably, however, while the same metrics did not detect differences between diseases in any individual center for HWP (C) and only for about half of the centers for TSI (B) and peak power (F), there were substantial differences within diseases for the latter two. Hence, standard characteristics did not reliably differentiate tremor diseases in a generalizable manner. [Color figure can be viewed at wileyonlinelibrary.com]

We first assessed established tremor characteristics to benchmark differentiation accuracy. To this end, we assess their consistency and generalizability within and between diagnoses and across centers for rest and postural recordings (Tables S3 and S4). This revealed that, in general, differences between ET and PD were more prominent in rest recordings (Fig. 2): tremor AUC, peak frequency, and FWHM failed to consistently differentiate between the conditions across comparisons, while TSI, HWP, and peak power differentiated successfully between diseases on a group level. However, this was not the case for each individual center with relevant differences within cohorts (Fig. 2B, C, F).

Taken together, TSI and peak power provided overall differentiation between diseases, with their combination outperforming each individual one, to reach a classification accuracy for triaxial data of 70.5% (80.8% specificity and 57.6% sensitivity; Table 1). This dropped to 63.9% (64.5% specificity and 63.3% sensitivity) when the monoaxial data were included. However, none of the standard characteristics provided a reliable and generalizable means of differentiation across all cohorts.

**TABLE 1:** Metrics of machine‐learning‐based classification in comparison with best‐performing established tremor characteristics

Parameter	ML model %	TSI %	Peak power %	TSI + peak power %
Accuracy	81.1	70.4	68.9	70.5
Sensitivity	86.4	70.8	68.7	57.6
Specificity	76.7	70.2	69.0	80.8
PPV	75.0	57.6	55.9	70.8
NPV	87.5	80.8	79.4	70.2

Abbreviations: ML, machine learning; TSI, tremor stability index; PPV, positive predictive value; NPV, negative predictive value.

### Differentiation Using Feature‐Based ML

#### Optimizing Recording Settings

To overcome the limitations inherent in targeted analyses, we explored the full spectrum of 7729 time series features identified by hctsa. In general, the combination of several best‐performing features improved classification accuracy up to 10 features (Fig. S1), but not beyond (Fig. S2). First, we aimed to determine the optimal parameters for supervised ML (Fig. S1A–C). This evaluation was based on the monocentric exploration dataset with the highest fidelity triaxial recordings (Graz) to mitigate any center‐specific biases in recording SOPs. Linear classifiers (support vector machine [SVM] up to 86.1% classification accuracy, logistic regression classifier up to 82.8%) outperformed tree‐based approaches such as random forest, particularly when integrating multiple features (Fig. S3A). Again, rest recordings consistently yielded higher classification accuracies (above 84%; Fig. S3B). In assessing the influence of the number of accelerometer axes on classification accuracy, we contrasted monoaxial recordings (Kiel) against both isolated single/z‐axis time series and vector amplitude sums from the same triaxial recordings (Graz). In this, the vector amplitude sums of the triaxial accelerometer data outperformed single‐axis data by approximately 13% (maximum accuracy of 86.1% vs. 73.8%) and monoaxial recordings by 16% (86.1% vs. 70.0%; Fig. S3C). Hence, we selected SVM and the vector amplitude sum of three axes as the ideal recording combination and tested for combinations of up to 10 features in all subsequent analyses on the *final data* dataset.

We next compared tremor recordings between ET and PD within the exploratory cohort. Adopting the approach utilized in establishing the TSI,^28^ we first compared ET *postural* versus PD *rest* recordings, despite the bias of contrasting different etiology‐specific postures (Fig. S3D). This yielded classification accuracies as high as 90.4% for London and 89.0% for Graz. We subsequently compared ET versus PD postural recordings (Fig. S3E), as well as ET versus PD rest recordings (Fig. S3F), achieving accuracies of up to 86.3% (Graz) and 84.6% (London): again, rest tremor recordings provided more effective differentiation than postural recordings.

### Identification of General Classifiers to Differentiate ET from PD Tremor

In order to identify features that reliably distinguish between ET and PD tremor signals across different centers we analyzed all available triaxial rest recordings (exploratory cohort consisting of 59 ET and 73 PD) for both non‐normalized as well as normalized time series. This resulted in nine amplitude‐dependent and four amplitude‐independent features (Table S3), each achieving a univariate classification accuracy >75% for differentiating ET from PD tremor. *MF_GARCHfit* emerged as the individual feature with the highest univariate classification accuracy.

**FIG. 3:**
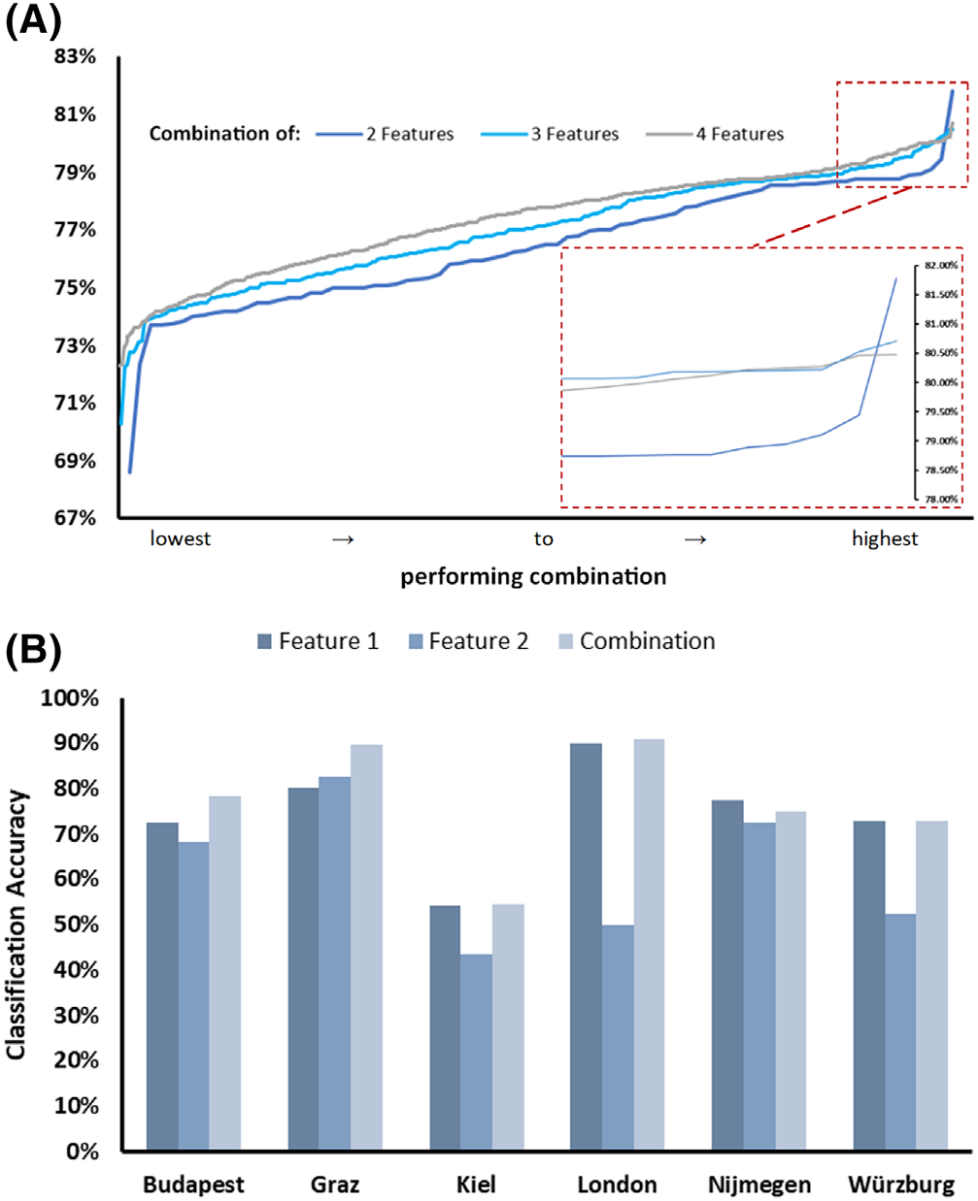
Assessment of combined, disease‐ and position‐specific tremor features. (A) To identify the ideal feature combination for disease differentiation, the performance of random combinations of two, three, or four of all the top‐performing univariate features were assessed and arranged in ascending order (worst to best classification) by their combined classification accuracy. Notably, combining more than two features did not improve overall classification accuracy. (B) The two features best performing in combination (feature 1: ‘MF_GARCHfit_ar_P1_Q1.stde_normksstat’, feature 2: ‘MF_hmm_CompareNStates_06_24.maxLLtrain’) showed consistently good accuracy across triaxial datasets, despite feature 2 individually performing worse on the London dataset. Performance on monoaxial data was consistently lower. [Color figure can be viewed at wileyonlinelibrary.com]

To determine the best‐performing combination of features across centers, we next tested all possible combinations of two, three, and four of the 13 features identified for their combined classification accuracy on all exploratory rest recordings. The paired combination of *MF_GARCHfit* (non‐amplitude dependent) and *MF_hmm_CompareNStates* (amplitude‐dependent feature) emerged as the optimal choice, yielding a combined classification accuracy of 81.8%. The addition of more than two features did not further improve overall performance (Fig. 3A). Across centers, the combination of both features consistently outperformed each feature alone (Fig. 3B). Notably, *MF_hmm_CompareNStates* exhibited lower accuracy in the London dataset, but this did not significantly affect the overall combined accuracy. Consistent with the standard tremor characteristic analyses, these features showed more than 25% lower classification accuracy when applied to monoaxial recordings (Kiel). In the validation datasets, the combination of *MF_GARCHfit* and *MF_hmm_CompareNStates* resulted in a combined classification accuracy of 72.5% (Nijmegen) and 72.7% (Wuerzburg). To quantify the higher information content in assessing the clinically more affected hand, repeating this analysis using time series from one randomly chosen hand per patient reduced the accuracy to 74.2% (−7.6%) in the trainset, 65% (−7.5%) for Nijmegen, and 68.2% (−4.3%) for Wuerzburg.

### Position‐Specific Tremor Features

In order to identify position‐specific tremor features irrespective of tremor diagnosis, we compared 132 rest and 132 postural recordings from all triaxial ET and PD recordings from our exploratory cohort. In comparison with the found poor accuracies for standard characteristics (49.2% for TSI; Table S6), ML‐based feature differentiation identified several features with excellent classification accuracy (Table S7). The combination of the best‐performing features yielded a classification accuracy of 99.6%, which successfully validated in the validation dataset (97.4%).

### Interpretation of Disease‐Defining Tremor Features

**FIG. 4:**
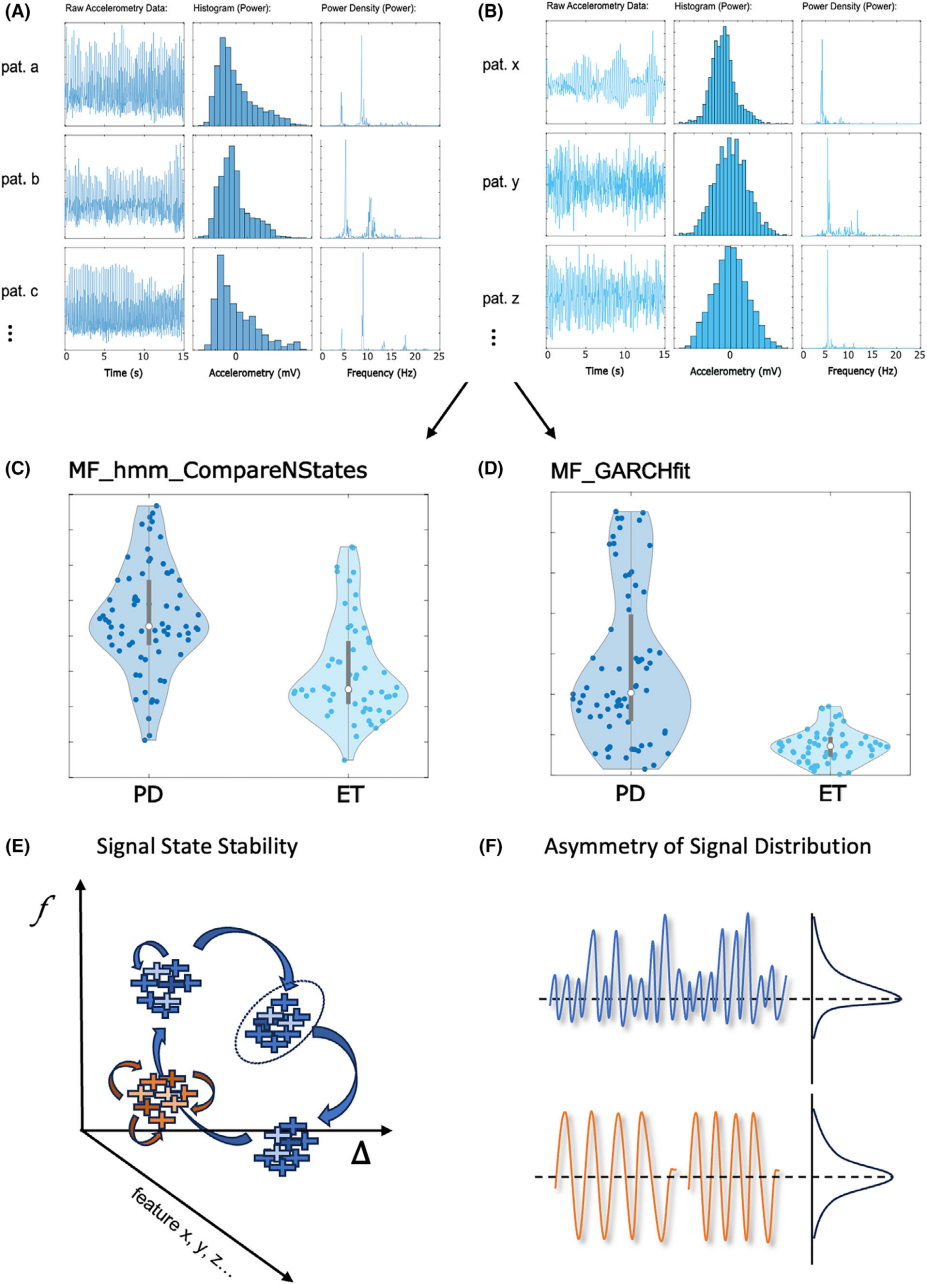
Interpretation of disease‐defining signal features. The two best‐performing features capture different aspects of the time series signal: exemplary data plots from raw accelerometry, power histogram, and power density of randomly selected (A) Parkinson's disease (PD) and (B) essential tremor (ET) patient recordings graphically illustrate different signal characteristics. Violin plots capture the observed distributions and group differences in calculated feature values between PD and ET recordings for features (C) ‘MF_hmm_CompareNStates’ and (D) ‘MF_GARCHfit’. The mechanistic translation of identified features is based on the interpretation of the underlying mathematical feature function, and graphically illustrated (E, F): MF_hmm_CompareNStates (E) assesses the position of consecutive time series signal points (illustrated as colour‐coded crosses) in a multidimensional feature space. Signal positions change over time, either within a ‘signal state’ (dotted circle), marking a period of relative signal stability, or between signal states, marking a change to another period of relative signal stability. ‘MF_hmm_CompareNStates’ evaluates the quality of fitting ≥2 states to the data. Low quality fits of the Hidden Markov Model (HMM) suggest that the data cannot be well explained with ≥2 states, suggesting that the signal stays within a singular signal state (red). Higher‐quality fitting of the HMM suggests that the signal naturally varies between several relatively stable signal states (blue). From the examined tremor data, ET tremor signals stay within a singular signal state, whereas PD tremor signals have a higher likelihood to change between ≥2 stable signal states. ‘MF_GARCHfit’ (F) examines how far histogram‐converted regular oscillations of different frequencies follow a Gaussian distribution (red) or deviate from it (blue). The latter occurs, for example, if a regular/symmetrical signal at a certain frequency is combined with asymmetrical oscillations. Higher values in this skewness metric relate to more prevalent asymmetries in the frequency distribution, as evident in PD tremor signals, whereas ET tremor signals show much lower levels of asymmetry. [Color figure can be viewed at wileyonlinelibrary.com]

To gain a more mechanistic understanding of the defining differences between ET and PD tremor, we interpreted the underlying mathematics of the identified features and related it to more intuitive accelerometry data and feature distributions (Fig. 4A–D).

Amplitude‐dependent *MF_hmm_CompareNStates* assesses the dynamic temporal evolution of a time series signal in a multidimensional feature space (Fig. 4A, C). Over time, signal characteristics can remain relatively stable, that is, within a stable ‘signal state’, or change between several ‘signal states’ (Fig. 4E). The calculation of this feature relies on a hidden Markov model as measured using log‐likelihood, namely fitted to the position of signal states, with the number of identified signal states < or ≥2 states defining the feature value. ET feature values show a poor fit to ≥ 2 states, indicating a higher probability that the signal remains within a singular signal state throughout the recording. Conversely, PD data fall clearly into several, separable but stable, generative states.

Amplitude‐independent *MF_GARCHfit* correlates with the skewness of the data, as illustrated by histogram plots (Fig. 4B, D). Specifically, its value increases with deviations from a Gaussian distribution, that is, if the relative contributions of certain acceleration signals are asymmetrically distributed (Fig. 4B). Such cases of higher signal skewness are particularly evident in PD tremor time series (Fig. 4D). This asymmetry relates to signals formed by a combination of oscillations, one or more of which are characterized by a direction‐specific acceleration, creating an asymmetric signal distribution (Fig. 4F). With higher feature values present in PD signals, we interpret this as a likely correlate of the more asymmetrical accelerations evident in PD rest tremor (eg, either the pill‐rolling phenomenon, or well‐known sudden fluctuations in hand tremor movements).

While *MF_hmm_CompareNStates* therefore quantifies signal variability, a trait somewhat similarly addressed by the TSI, *MF_GARCHfit* exploits conceptual signal properties previously not captured by any standard tremor characteristic.

### Stability of Features

To assess the influence of recording length, we calculated the classification accuracy of the best‐performing combined features *MF_GARCHfit* and *MF_hmm_CompareNStates* on increasingly shortened segments of the same time series from our exploratory cohort (59 ET, 73 PD; Fig. S3), revealing higher accuracy with longer time series data.

## Discussion

This work establishes multicenter feature‐based ML as a powerful novel method for unbiased tremor analysis and allows reliable, valid, and generalizable differentiation of the two most prevalent tremor disorders, as well as their mechanistic exploration.

### Standard Tremor Characteristics Provide No Reliable Differentiation Between ET and PD Tremor

This study represents the largest multicenter tremor accelerometry investigation to date.^28^, ^39^, ^41^ Our findings contribute significantly to understanding cross‐center variability in tremor characteristics, revealing notable differences between patient cohorts from different centers. While our study design does not allow to pinpoint the exact causes of this heterogeneity – be it population, recruitment, or diagnostic bias – it provides a comprehensive overview of signal variability in ET and PD. It is well‐acknowledged that patient presentations at tertiary referral centers differ from those in general neurology practices, but we were surprised about the heterogeneity even between academic centers. Our results highlight that none of the previously established tremor characteristics perform reliably across populations and centers – necessitating the discovery of novel, generalizable metrics. In turn, this means that any future metric needs to validate on data from multiple cohorts in order to capture real‐world variability.

An observation from our data is that stratification performs better with rest than with postural recordings. Although not surprising – clinically ET typically does not manifest with rest tremor, except in the case of ET‐plus^18^ – this observation was consistent across classic and ML‐based classification approaches. This study cannot provide insights into the signal characteristics of ET‐plus, while published first evidence suggests barely discernible signal differences.^51^

### Triaxial Accelerometry is Superior to Monoaxial Measurements

Historically, studies combined accelerometry with electromyography (EMG) to differentiate between the mechanical, mechanical‐reflex, and central component of tremor.^27^ Significant advances in tremor analysis^28^, ^31^ focused on accelerometer data^33^, ^34^ – while previously monoaxial,^26^, ^52^, ^53^ triaxial accelerometers are increasingly employed^28^, ^33^, ^45^, ^46^, ^54^ and recommended.^27^ Our results on accelerometer dimensionality are consistent: monoaxial recordings inherently reduce the information content of the three‐dimensional tremor signal, substantially affecting downstream signal analysis, resulting in consistently worse performance, arguing against future single‐axis analyses.

### Feature‐Based ML – An Unbiased Method for Tremor Analysis

The basic principle of ML is to “learn” patterns in complex data without human interference. This aims to overcome limitations of previous studies by applying a narrow set of variables or predefined, hypothesis‐driven characteristics.

Our study firmly establishes feature‐based ML as an unbiased, generalizable method for tremor analysis. Based on the entirety of mathematical features extracted from time series data,^44^ this methodology incorporates the maximum available information content from high‐fidelity movement recordings. As previous efforts,^28^, ^31^ feature‐based ML is based on movement characteristics only, not taking additional clinical information into account, which is often of paramount importance in daily practice. We demonstrate that this technique nevertheless can effectively categorize tremor data by disease entity or indeed recording position. Although many hctsa methods assume stationarity – a limitation given the inherently non‐stationary nature of tremor signals – we found that key dynamic features, such as fluctuations and state transitions within a single recording, were still robustly captured, allowing meaningful differentiation between tremor types despite this constraint.

Advances in artificial intelligence are taking shape in tremor research.^33^ The true strength of ML in this context is, however, only realized when paired with large, ideally, multicenter datasets, representing the physiological signal variability within a condition. Our findings on ET and PD are derived from a multicenter dataset with slightly inconsistent recording settings, limiting the vulnerability towards center‐, clinician‐, position‐, sensor‐placement‐, and equipment‐related bias. While possibly lowering our approach's calculated accuracy, these inconsistencies strengthen the model's generalizability to heterogeneous real‐world data. The rigorous use of cross‐ and external validation effectively minimizes the theoretical risk of overfitting. We anticipate that the defining features and resulting precision of ML‐based analyses will increase with larger and more clinically diverse sample sets, and we therefore recommend future international collaboration in this area. Nonetheless, our work demonstrates that ML‐based differentiation between tremor disorders is not only feasible and superior to previous metrics but can also aid our understanding of the underlying conditions.

### Deductions on Pathophysiology

The identification of features describing the signal state stability and asymmetry of signal distribution as the defining characteristics discerning ET from PD tremor are instructive of the underlying dynamics and origin of the two tremor disorders.

Parkinsonian rest tremor is known to show spontaneous fluctuations in amplitude but less frequency over time.^55^, ^56^ This relatively broad frequency‐amplitude tolerance, contrasting with a much narrower tolerance in ET, is summarized in the concept of TSI,^28^ deducted from the spontaneous evolution of frequency fluctuations and the observation of different amplitude modulation effects despite significant frequency entrainment by brain stimulation.^55^ Equally, tremor amplitude in PD shows a “waxing and waning” quality, and spontaneous amplitude fluctuations are preceded by increased cerebral integration several seconds before “waxing” episodes.^57^ The detection of two or more relatively stable signal states in PD tremor time series is a strong indication of either several discrete oscillatory pacemakers, leading the rest tremor circuitry in turn, or several dynamic activity states of involved structures. The observation that the signal fluctuates between a discrete number of stable states points towards a dynamic interplay between involved nodes. Theoretically this could be due to the pacemaker activity undulating back and forth between structures, or alternatively effects of drifting phase alignments in the rhythmicity between core oscillatory pacemakers.

Similarly, an asymmetric signal distribution in PD tremor supports the current view of at least two central tremor oscillation pacemakers in PD, likely situated in the basal ganglia and the cerebello‐thalamo‐cortical motor loop.^58^ The currently prevailing theory on the origin of parkinsonian rest tremor, the *dimmer‐switch model*, posits that this origin stems from the basal ganglia (globus pallidus and subthalamic nucleus [STN]), while the cerebello‐thalamo‐cortical loop modulates tremor amplitude.^14^ Microelectrode recordings detected oscillations at tremor frequency not only in the ventral intermediate thalamic (VIM) nucleus, but also the STN and the internal pallidum (GPi).^59^, ^60^, ^61^, ^62^ Further, different frequency bands within the same anatomical structure (STN) have been shown to have opposing effects on PD rest tremor, representing additional, functionally discrete central oscillators.^62^, ^63^ Our data hence provide additional evidence to support an interaction between several central oscillators in the generation of PD rest tremor, resulting in several discrete but stable signal states.

Conversely, the presence of a singular stable signal space with a symmetrical signal distribution in our data is compatible with either a singular oscillator or a much more tightly connected net of oscillators in ET. Previously, the narrower TSI has been interpreted as a result of stronger coupling between the central elements involved in tremor generation.^28^, ^55^ Although our data cannot provide the anatomical origin of such activity, it strongly supports this hypothesis.

### Context and Implications

Tremor disorders are clinical diagnoses. Historically, classification systems have developed based on the clinician's interpretation of phenotypes.^16^, ^17^, ^18^, ^64^ This classification framework is rooted in the historical routine of deducting clinico‐pathological correlation from the astute observation and interpretation of subtle movement characteristics.^65^ However, the diagnostic yield of this approach is known to be limited, particularly in the case of ET.^66^, ^67^ Recent efforts to refine these classifications have led to the reconceptualization of ET as a clinical syndrome,^18^ acknowledging the diagnostic uncertainty prevalent in many tremor cases.^68^ Whilst criticized,^69^ this new classification – likely yet another intermediate step in the continuing evolution of the clinical concept ET^70^ – is taking clinical soft signs, known to have low clinimetric accuracy and poor interrater reliability,^68^, ^71^, ^72^, ^73^ into account.

Despite these efforts, there remains no universally accepted biomarker to aid objective tremor classification, shrouding results from decades of research into the epidemiology, pathophysiology, and genetics of ET, in a cloud of relative ambiguity.^34^, ^74^, ^75^, ^76^, ^77^ This a priori uncertainty must be considered when interpreting the results of studies like ours that use the current clinical diagnostic gold standard as reference.

Notwithstanding the value of clinical phenotyping, our study lays the foundation for a more objective, generalizable approach to describing and characterizing tremor. Stratification by specific movement characteristics has already proven to have relevant therapeutic implications, exemplified by the results from phase‐locked invasive and non‐invasive brain stimulation in several tremor disorders that have shown that rhythmic tremor following a sinusoidal activity is more amenable to phase‐specific intervention.^44^, ^78^, ^79^ There is therefore emerging evidence to support the notion that not the clinical diagnosis per se, but the presence of particular movement characteristics, may predict the response to certain therapeutic interventions^67^, ^80^ – an emerging concept possibly not limited to the effect of electrical stimulation.

Certainly, future studies including larger datasets will achieve a more accurate performance and we acknowledge that the included data represent a population of predominantly European‐Caucasian descent only. As outliers disproportionally influence ML results,^33^ carefully curated datasets are paramount.

## Conclusions

This study establishes feature‐based ML as a novel analytical tool for unbiased tremor analysis, outperforming previous electrophysiology metrics for tremor stratification, and offering a means for hypothesis‐free exploration of complex time series signals. Future studies must incorporate large, diverse samples from multiple centers and ideally various genetic backgrounds in order to establish truly generalizable tremor classification.

## Author Roles

(1) Research Project: A. Study Design, B. Data Collection; (2) Statistical Analysis: A. Design, B. Execution, C. Review and Critique; (3) Manuscript Preparation: A. Writing of the First Draft, B. Review and Editing.

V.H.: 1B, 2B, 3A.

V.S.: 1B, 2B, 3B.

J.F.M.‐R.: 1B.

P.S.: 1B.

G.T.: 1B.

L.K.: 1B.

J.R.: 1B.

S.P.: 1B.

F.G.: 1B.

E.M.: 1B.

A.L.: 1B.

P.M.: 1B, 3B.

R.C.H.: 1B, 3B.

K.P.B.: 3B.

J.V.: 3B.

R.P.: 1A, 2B, 3B.

S.R.S.: 1A, 2A, 3A.

## Supporting information


**Supplementary Fig. S1. Accuracy of unbiased feature‐based supervised tremor classification depends on machine learning algorithm, recording position, sensor‐dimensionality, and number of features**. To explore which fundamental analytical settings are best suited to differentiate essential tremor (ET) and Parkinson's disease (PD) tremor we systematically examined the effects of (**A**) machine learning algorithm, (**B**) recording position, (**C**) sensor‐dimensionality (monoaxial vs. isolated single axis vs. vector amplitude sum), and number of combined best‐performing features on classification accuracy. For the differentiation between PD and ET we used the comparison between (**D**) PD rest vs. ET postural, (**E**) PD vs. ET postural, as well as (**F**) PD vs. ET rest recordings. All data are based on hctsa features extracted from 15 s segments of tremor accelerometer recordings (down‐sampled to 100 Hz) from the more severely affected hand, and support vector machine algorithm (**B–F**) with an ascending number of combined features on the x‐axis.

**Supplementary Fig. S2. Changes of accuracy of tremor classification with ever increasing number of features**. As an extension of feature exploration (Fig. 3), increasing number of feature combinations were explored to identify the best‐suited feature combinations to differentiate essential tremor (ET) and Parkinson's disease (PD) tremor. Extending the feature count >10 did not improve overall differentiation accuracy for the most relevant settings algorithm, position, and dimensionality. All data are based on hctsa features extracted from 15 s segments of tremor accelerometer recordings (down‐sampled to 100 Hz) from the more severely affected hand, and support vector machine ML algorithm (**B–F**) with an ascending number of combined features on the x‐axis.

**Supplementary Fig. S3. Differentiation accuracy depends on signal length**. The stability and duration‐dependence of features were examined using progressively longer segments of the same time series from the exploratory cohort (59 essential tremor [ET], 73 Parkinson's disease [PD]). Differentiation accuracy gradually increased with longer time series.

**Table S1. Clinical and demographic details of participants**.
**Table S2. Center‐specific accelerometry specifications**.
Table S3. Standard tremor characteristics – rest recordings.
**Table S4. Standard tremor characteristics – postural recordings**.
**Table S5. List of individually best‐performing time series features to differentiate essential tremor (ET) from Parkinson's disease (PD) rest tremor recordings; features 1–9 are amplitude‐dependent and features 10–13 are amplitude‐independent**.
**Table S6. Metrics of machine‐learning‐based classification for comparing rest from postural tremor recordings in comparison with established tremor characteristics**.
**Table S7. Top 10 individually best‐performing tremor features to differentiate rest from postural tremor recordings irrespective of clinical diagnoses (essential tremor [ET] or Parkinson's disease [PD]); features are amplitude‐dependent**.

## Data Availability

The data that support the findings of this study are available from the corresponding author upon reasonable request.
