# Supplementary figures and images for: Phenotypical Differentiation of Tremor Using Time Series Feature Extraction and Machine Learning
Source: Mov Disord. 2025 Sep 5;40(12):2628–40. doi: 10.1002/mds.70032 (PMC12710121; doi:10.1002/mds.70032)

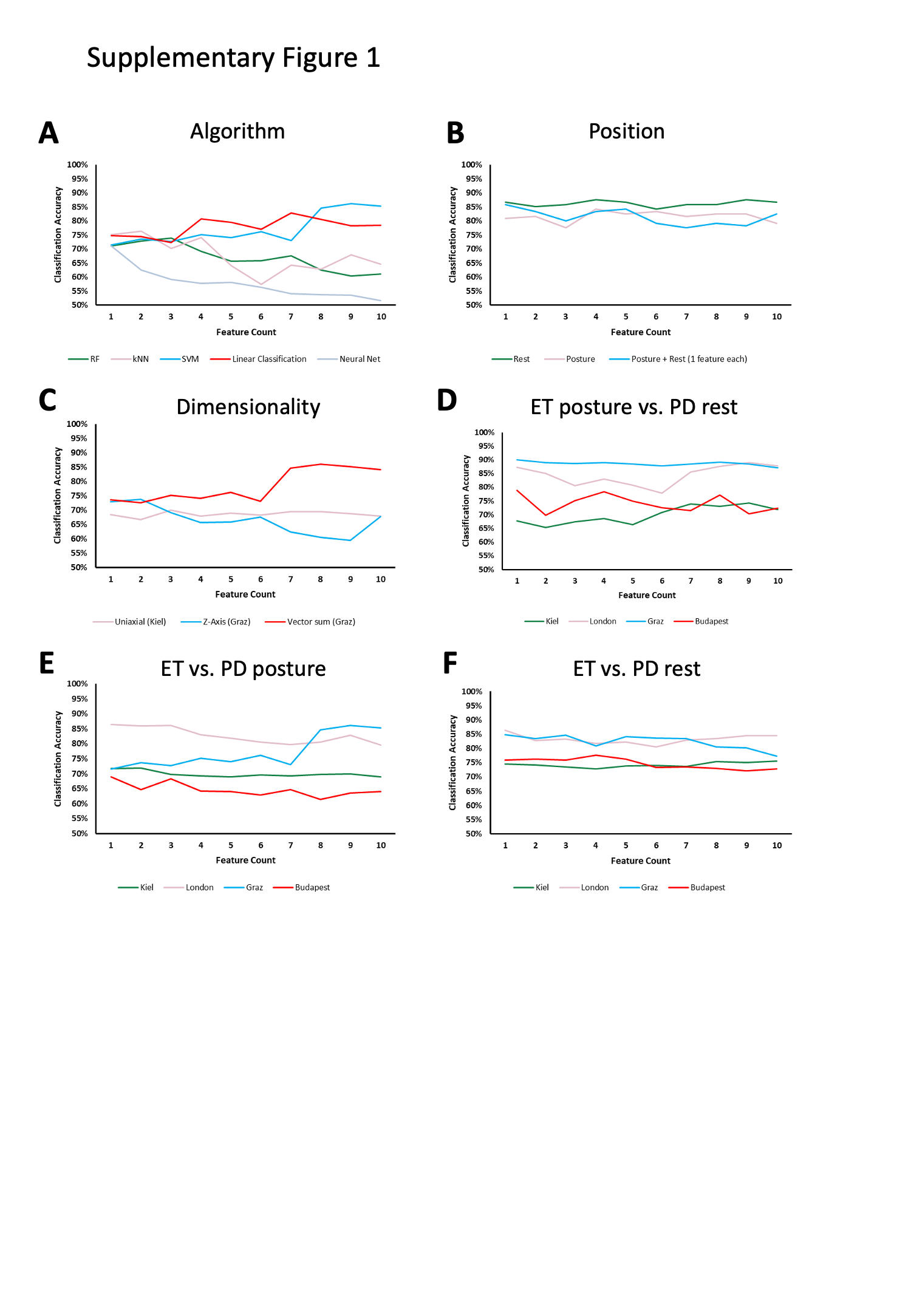

Supplement: Supplementary file 1 — Supplementary Fig. S1. Accuracy of unbiased feature‐based supervised tremor classification depends on machine learning algorithm, recording position, sensor‐dimensionality, and number of features. To explore which fundamental analytical settings are best suited to differentiate essential tremor (ET) and Parkinson's disease (PD) tremor we systematically examined the effects of (A) machine learning algorithm, (B) recording position, (C) sensor‐dimensionality (monoaxial vs. isolated single axis vs. vector amplitude sum), and number of combined best‐performing features on classification accuracy. For the differentiation between PD and ET we used the comparison between (D) PD rest vs. ET postural, (E) PD vs. ET postural, as well as (F) PD vs. ET rest recordings. All data are based on hctsa features extracted from 15 s segments of tremor accelerometer recordings (down‐sampled to 100 Hz) from the more severely affected hand, and support vector machine algorithm (B–F) with an ascending number of combined features on the x‐axis. [file MDS-40-2628-s002.tiff]

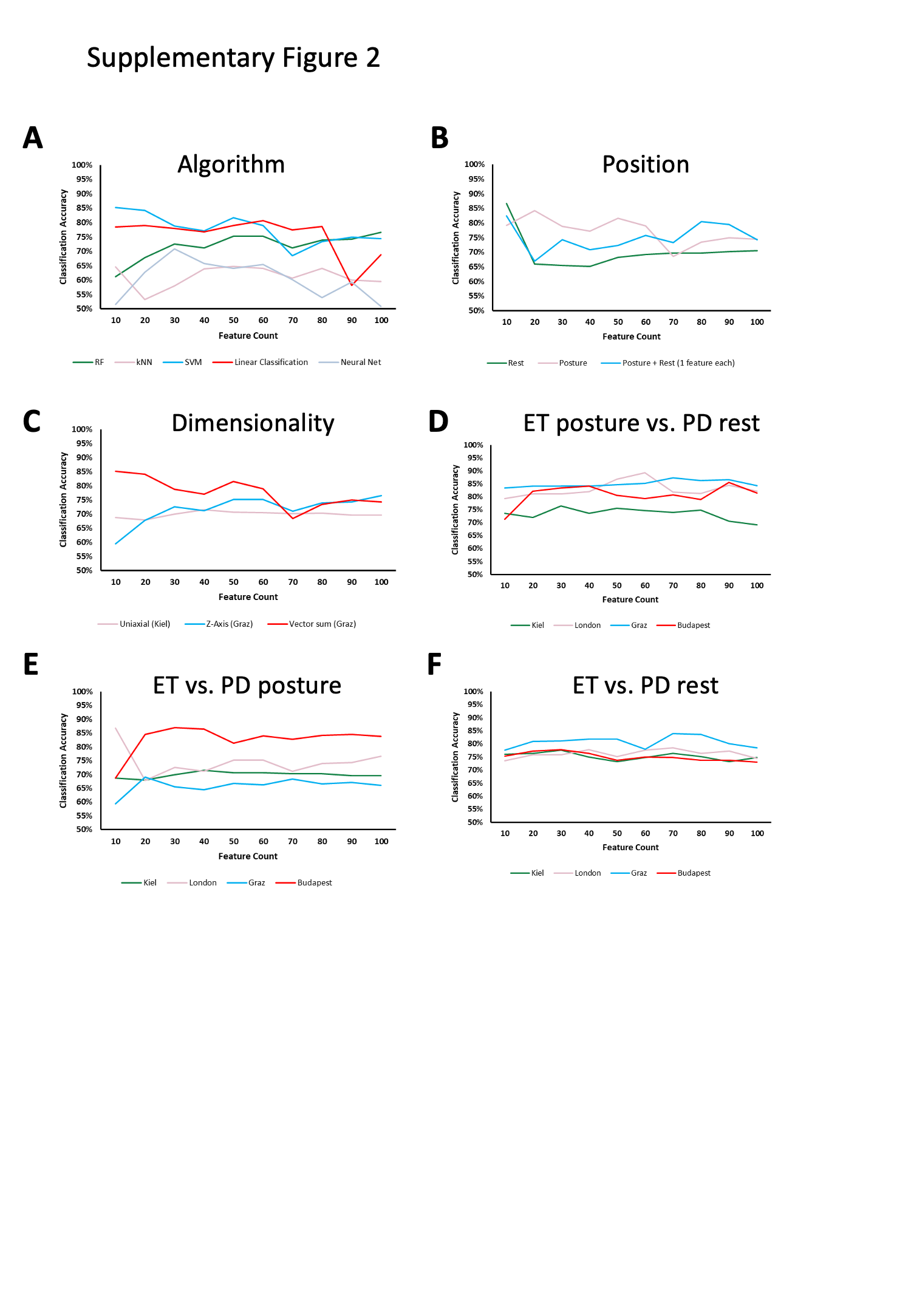

Supplement: Supplementary file 2 — Supplementary Fig. S2. Changes of accuracy of tremor classification with ever increasing number of features. As an extension of feature exploration (Fig. 3), increasing number of feature combinations were explored to identify the best‐suited feature combinations to differentiate essential tremor (ET) and Parkinson's disease (PD) tremor. Extending the feature count >10 did not improve overall differentiation accuracy for the most relevant settings algorithm, position, and dimensionality. All data are based on hctsa features extracted from 15 s segments of tremor accelerometer recordings (down‐sampled to 100 Hz) from the more severely affected hand, and support vector machine ML algorithm (B–F) with an ascending number of combined features on the x‐axis. [file MDS-40-2628-s004.tiff]

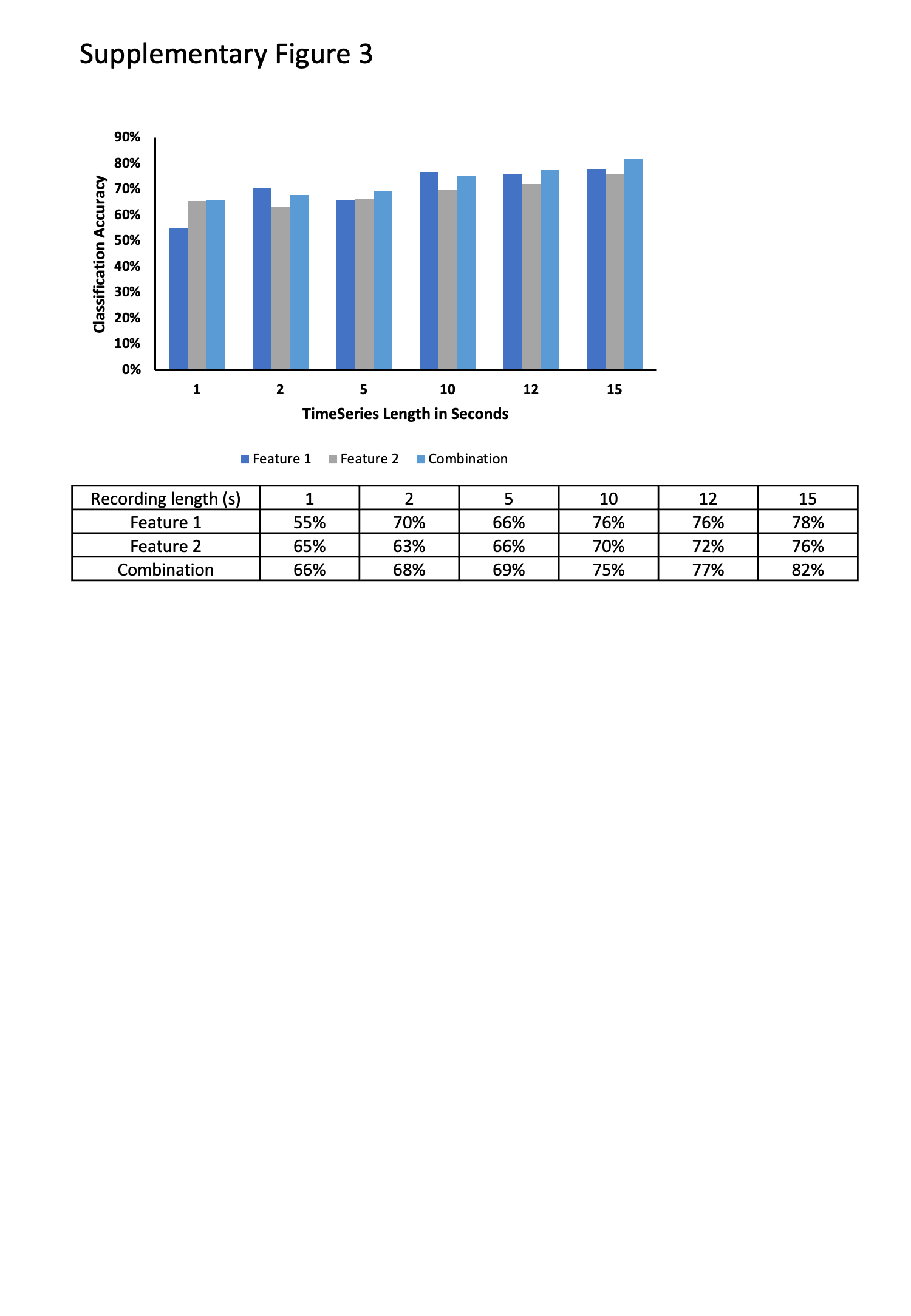

Supplement: Supplementary file 3 — Supplementary Fig. S3. Differentiation accuracy depends on signal length. The stability and duration‐dependence of features were examined using progressively longer segments of the same time series from the exploratory cohort (59 essential tremor [ET], 73 Parkinson's disease [PD]). Differentiation accuracy gradually increased with longer time series. [file MDS-40-2628-s003.tiff]
