# Supplementary material for: Phenotypical Differentiation of Tremor Using Time Series Feature Extraction and Machine Learning
Source: Mov Disord. 2025 Sep 5;40(12):2628–40. doi: 10.1002/mds.70032 (PMC12710121; doi:10.1002/mds.70032)
Supplement: Supplementary file 4 — Table S1. Clinical and demographic details of participants. Table S2. Center‐specific accelerometry specifications. Table S3. Standard tremor characteristics – rest recordings. Table S4. Standard tremor characteristics – postural recordings. Table S5. List of individually best‐performing time series features to differentiate essential tremor (ET) from Parkinson's disease (PD) rest tremor recordings; features 1–9 are amplitude‐dependent and features 10–13 are amplitude‐independent. Table S6. Metrics of machine‐learning‐based classification for comparing rest from postural tremor recordings in comparison with established tremor characteristics. Table S7. Top 10 individually best‐performing tremor features to differentiate rest from postural tremor recordings irrespective of clinical diagnoses (essential tremor [ET] or Parkinson's disease [PD]); features are amplitude‐dependent. [file MDS-40-2628-s001.docx]

**Phenotypical differentiation of Tremor using time series feature extraction and machine learning**

**Table S1 Clinical and demographic details of participants.**

|  | ET | | | | | | PD | | | | | | All (mean ± SD) | | |
| --- | --- | --- | --- | --- | --- | --- | --- | --- | --- | --- | --- | --- | --- | --- | --- |
|  | **Graz** | **Budapest** | **London** | **Kiel** | **Nijmegen** | **Wuerzburg** | **Graz** | **Budapest** | **London** | **Kiel** | **Nijmegen** | **Wuerzburg** | **ET** | **PD** | **stat** |
| N= | 17 | 36 | 6 | 99 | 9 | 21 | 21 | 47 | 5 | 99 | 31 | 23 | 89 (188 incl. Kiel) | 127 (226  incl. Kiel) | / |
| Age | 60.6 **±** 19.7  (1 missing) | 67.6 **±** 13.0 | 55.0 **±** 14.5 | n.a. | 59.0 **±** 14.9 | 59.0 ± 14.67 | 63.4 **±** 11.9 | 63.7 **±** 10.1 | 73.6 **±** 10.6 | n.a. | 63.0 **±** 7.9 | 62.56 ± 10.33 | 62.51 **±** 15.74  (1 missing) | 63.57 **±** 10.2 | *P*=0.55 |
| Sex (% f) | 21.4%  (3 missing) | 58.3% | 50.0% | n.a. | 11.1% | 23.8% | 45.0%  (1 missing) | 42.6% | 20.0% | n.a. | 12.9% | 17.4% | 37.5% (3 missing) | 30.1%  (1 missing) | *P*=0.23 |
| Age at onset | 37.8 **±** 21.8  (3missing) | 51.5 **±** 20.5 | 39.3 **±** 15.9 | n.a. | 17.0 **±** 15.3 | 23.28 ± 17.09 | 56.9 **±** 12.8  (1 missing) | 57.1 **±** 13.6 | 62.6 **±** 7.4 | n.a. | 56.0 **±** 8.9 | 56.43 ± 10.73 | 37.99 **±** 23.29  (3 missing) | 57.02 **±** 11.79  (1 missing) | *P*=0.1 * 10^-5^  (P<0.05) |
| Tremor duration | 20.8 **±** 23.5  (3 missing) | 16.0 **±** 15.0 | 15.7 **±** 9.4 | n.a. | 42.0 **±** 17.9 | 35.24 ± 19.97 | 6.7 **±** 5.8  (1 missing) | 5.3 **±** 4.4 | 11 **±** 8.3 | n.a. | 6 **±** 3.0 | 8.30 ± 11.00 | 24.13 **±** 20.44  (3 missing) | 6.54 **±** 6.45  (1 missing) | *P*=1.1 * 10^-11^  (P<0.05) |
| CRST | 24.4 ± 9.9  (3 missing, $) | 39.1 **±** 15.9 ($) | 17.8 **±** 10.1  (1missing, $) | n.a. | 37.4 ± 14.7 (#) | 31.76 ± 9.58 ($) | / | | | | | | $ 32.96 ± 14.57  (4 missing)  # 37.4 ± 14.7 | / | / |
| MDS-UPDRS part III | / | | | | | | 29.3 ± 15.8  (10missing) | 20.5 **±** 17.1 | 43.4 ± 13.7 | n.a. | 44.0 **±** 11.7  (1missing) | 34.39 ± 11.71 | / | 31.10 **±** 17.55  (11 missing) | / |

**CRST=Fahn-Tolosa-Marin scale; MDS-UPDRS = Movement Disorder Society-United Parkinson´s disease rating scale; / = not applicable; n.a. = not available; $ = CRST items 1-21; # = CRST items 1-14;**

**Table S2 Centre-specific accelerometry specifications.**

|  | **Graz** | **Budapest** | **London** | **Kiel** | **Nijmegen** | **Wuerzburg** |
| --- | --- | --- | --- | --- | --- | --- |
| **Accelerometer** | Tri-axial accelerometer (Biometrics ACL300 + amplifier K800, sensitivity ± 100 mV/g) | Tri-axial accerelometer (Kinesia quatitative motor assessment system; Great Lakes Neurotechnologies, sensitivity ± 4.5mV/g) | Tri-axial accelerometer (MMA7361, Freescale Semiconductor, Inc.; sensitivity ± 800 mV/g) | Mono-axial accelerometer (Jäger-Tönnies GmbH/ VIASYS Healthcare Inc., sensitivity ± 50 mV/g) | Tri-axial accelerometer (Brain Products; sensitivity ± 1450 mV/g) | Tri-axial accelerometer (Biometrics ACL500, sensitivity ± 100 mV/g) |
| **Sampling frequency** | 1000Hz | 128Hz | 500Hz | 800Hz | 5000Hz | 2000Hz |
| **Sensor placement** | Dorsum of middle phalanx, index ﬁnger | Dorsum of middle phalanx, index finger | Dorsum of proximal phalanx, middle finger | Dorsum of proximal phalanx, middle finger | Dorsum of the hand | Dorsum of proximal phalanx, middle finger |
| **Rest position** | 30s hands hanging freely from arm rest, supported lower arm | 15s hands hanging freely from arm rest, supported lower arm | 20s hands resting on ulnar edge on chair armrest / table | 60s hands hanging freely from arm rest, supported lower arm | 60s hands hanging freely from arm rest, supported lower arm | 30s hands hanging freely from arm rest, supported lower arm |
| **Posture position** | 30s arms/wrists outstretched at shoulder level, fingers prone slightly parted | 15s arms/wrists outstretched at shoulder level, fingers prone slightly parted | 20s arms/wrists outstretched at shoulder level, fingers prone slightly parted | 60s hands extended, prone, supported lower arm | 60s arms/wrists outstretched at shoulder level, fingers prone slightly parted | 30s arms/wrists outstretched at shoulder level, fingers prone slightly parted |

**Table S3 Standard Tremor Characteristics – rest recordings:**

|  | ET | | | | | | PD | | | | | | All (mean ± SD) | |
| --- | --- | --- | --- | --- | --- | --- | --- | --- | --- | --- | --- | --- | --- | --- |
|  | **Graz** | **Budapest** | **London** | **Kiel** | **Nijmegen** | **Würzburg** | **Graz** | **Budapest** | **London** | **Kiel** | **Nijmegen** | **Würzburg** | **ET** | **PD** |
| N= | 17 | 36 | 6 | 99 | 9 | 21 | 21 | 47 | 5 | 99 | 31 | 23 | 188 | 226 |
| AUC | 0.70±0.20 | 1.23±0.22 | 1.11±0.18 | 0.97±0.19 | 1.05±0.17 | 1.11± 0.20 | 1.02±0.25 | 1.06±0.22 | 1.10±0.23 | 0.91±0.24 | 0.90±0.22 | 0.88 ±0.30 | 1.02±0.24 | 0.95±0.25 |
| TSI | 2.18±1.00 | 1.51±0.88 | 1.70±0.79 | 1.51±0.77 | 1.09±0.58 | 1.39± 0.59 | 0.99±0.84 | 0.90±0.72 | 0.86±0.63 | 1.08±0.81 | 0.70±0.48 | 1.01± 0.85 | 1.54±0.82 | 0.97±0.77 |
| HWP | 0.03±0.04 | 0.07±0.04 | 0.06±0.04 | 0.07±0.06 | 0.09±0.04 | 0.14± 0.02 | 0.07±0.04 | 0.10±0.05 | 0.09±0.06 | 0.10±0.06 | 0.12±0.05 | 0.16± 0.03 | 0.08±0.06 | 0.10±0.06 |
| Peak Frequency | 7.17±2.98 | 6.44±2.37 | 6.09±6.10 | 4.14±2.43 | 5.88±0.61 | 6.67± 1.26 | 6.36±2.27 | 5.86±1.80 | 4.91±0.87 | 5.29±1.80 | 5.25±1.18 | 5.90± 0.91 | 5.28±2.81 | 5.55±1.73 |
| FWHM | 0.33±0.37 | 0.29±0.23 | 0.36±0.23 | 0.33±0.31 | 0.32±0.36 | 0.13± 0.06 | 0.33±0.28 | 0.49±0.66 | 0.39±0.41 | 0.42±0.44 | 0.41±0.40 | 0.14± 0.06 | 0.30±0.29 | 0.39±0.47 |
| Peak Power | 0.18±0.17 | 0.43±0.23 | 0.34±0.20 | 0.38±0.20 | 0.60±0.18 | 0.17± 0.05 | 0.48±0.26 | 0.58±0.20 | 0.56±0.23 | 0.53±0.24 | 0.62±0.19 | 0.19± 0.06 | 0.36±0.22 | 0.51±0.24 |

**Table S4 Standard Tremor Characteristics – postural recordings:**

|  | ET | | | | | | PD | | | | | | All (mean ± SD) | |
| --- | --- | --- | --- | --- | --- | --- | --- | --- | --- | --- | --- | --- | --- | --- |
|  | **Graz** | **Budapest** | **London** | **Kiel** | **Nijmegen** | **Würzburg** | **Graz** | **Budapest** | **London** | **Kiel** | **Nijmegen** | **Würzburg** | **ET** | **PD** |
| N= | 17 | 36 | 6 | 99 | 9 | 21 | 21 | 47 | 5 | 99 | 31 | 23 | 188 | 226 |
| AUC | 1.18±0.20 | 1.13±0.19 | 1.23±0.09 | 0.95±0.24 | 1.10±0.21 | 0.94±0.25 | 1.10±0.23 | 1.13±0.25 | 1.19±0.20 | 0.90±0.24 | 1.12±0.22 | 0.97±0.28 | 1.02±0.25 | 1.01±0.27 |
| TSI | 1.38±0.78 | 0.98±0.58 | 1.34±0.73 | 1.14±0.85 | 1.49±0.87 | 1.01± 0.62 | 1.52±1.03 | 1.17±0.90 | 0.80±0.49 | 1.05±0.83 | 1.07±0.61 | 1.21± 0.90 | 1.14±0.79 | 1.13±0.85 |
| HWP | 0.09±0.10 | 0.07±0.03 | 0.05±0.02 | 0.07±0.04 | 0.06±0.03 | 0.15± 0.03 | 0.05±0.03 | 0.10±0.06 | 0.09±0.04 | 0.10±0.07 | 0.08±0.03 | 0.15± 0.03 | 0.08±0.05 | 0.10±0.06 |
| Peak Frequency | 7.18±2.34 | 6.08±1.00 | 3.90±1.88 | 4.82±2.21 | 7.25±1.28 | 6.39±1.10 | 6.28±2.63 | 6.03±1.59 | 4.21±1.22 | 5.47±1.85 | 5.92±2.11 | 6.49±1.47 | 5.53±2.12 | 5.80±1.963 |
| FWHM | 0.55±1.10 | 0.20±0.08 | 0.27±0.09 | 0.28±0.29 | 0.17±0.06 | 0.14± 0.05 | 0.23±0.11 | 0.47±0.66 | 0.36±0.26 | 0.40±0.59 | 0.28±0.21 | 0.14± 0.06 | 0.27±0.41 | 0.36±0.51 |
| Peak Power | 0.41±0.20 | 0.53±0.17 | 0.39±0.11 | 0.50±0.25 | 0.58±0.27 | 0.19±0.06 | 0.35±0.18 | 0.50±0.16 | 0.56±0.25 | 0.54±0.23 | 0.50±0.17 | 0.18±0.05 | 0.46±0.25 | 0.47±0.23 |

**Table S5 List of individually best-performing time-series features to differentiate ET from PD rest tremor recordings; feature 1-9 are amplitude-dependent, feature 10-13 are amplitude-independent;**

| **Feature-number** | **Feature:** | **Univariate Classification Accuracy:** |
| --- | --- | --- |
| **Amplitude-dependent:** | | |
| 1046 | 'NL_DVV_3_100_2_50_10_default.trend' | 75.13% |
| 1169 | CO_AddNoise_1_quantiles_10.firstUnder50 | 75.13% |
| 1208 | 'CO_AddNoise_1_even_10.firstUnder75' | 75.13% |
| 2858 | EN_mse_1-10_2_015_diff1.meanch | 75.59% |
| 6581 | WL_dwtcoeff_db3_5.noisestd_l1 | 75.13% |
| 6713 | NL_MS_nlpe_2_mi.normp | 75.03% |
| 7584 | MF_hmm_07_3.LLtrainpersample | 75.79% |
| 7601 | MF_hmm_CompareNStates_06_24.meanLLtrain | 77.13% |
| 7602 | MF_hmm_CompareNStates_06_24.maxLLtrain | 75.69% |
| **Amplitude- independent:** | | |
| 7441 | MF_GARCHfit_ar_P1_Q1.stde_normksstat' | 77.79% |
| 7442 | MF_GARCHfit_ar_P1_Q1.stde_normp | 78.67% |
| 7498 | MF_GARCHfit_ar_P1_Q2.stde_normksstat | 77.23% |
| 7499 | MF_GARCHfit_ar_P1_Q2.stde_normp | 77.23% |

**Table S6 Metrics of machine-learning based classification for comparing rest from postural tremor recordings in comparison to established tremor characteristics.**

|  | **ML Model** | **AUC** | **TSI** | **HWP** | **Peak frequency** | **FWHM** | **Peak power** |
| --- | --- | --- | --- | --- | --- | --- | --- |
| **Accuracy** | 99.6% | 39.0% | 49.2% | 43.2% | 48.5% | 45.5% | 47.0% |
| **Sensitivity** | 99.2% | 48.5% | 59.1% | 43.2% | 64.1% | 43.9% | 38.6% |
| **Specificity** | 100% | 29.5% | 39.4% | 43.2% | 35.6% | 47.0% | 55.3% |
| **Positive predictive value** | 100% | 40.8% | 49.4% | 43.2% | 48.8% | 45.3% | 46.4% |
| **Negative predictive value** | 99.2% | 36.4% | 49.1% | 43.2% | 48.9% | 45.6% | 47.4% |

**Table S7 Top 10 individually best-performing tremor features to differentiate rest- from postural tremor recordings irrespective of clinical diagnoses (ET or PD); features are amplitude dependent;**

| **Feature Number:** | **Feature:** | **Univariate Classification Accuracy** |
| --- | --- | --- |
| 1036 | \| HT_DistributionTest_chi2logn5 \| \| --- \| | 100% |
| 1218 | \| CO_AddNoise_ac_quantiles_10.linfit_mse \| \| --- \| | 100% |
| 1256 | \| CO_AddNoise_ac_even_10.linfit_mse \| \| --- \| | 100% |
| 5270 | \| NL_TSTL_dimensions_50_ac_fnnmar.scr_bc_m2_meansqres \| \| --- \| | 99.6% |
| 7561 | SY_VarRatioTest_4_1.ratio | 99.2% |
| 7567 | SY_VarRatioTest_24682468_00001111.IIDperiodmaxpValue | 99.2% |
| 5975 | PP_Compare_spline64.olbt_s2 | 59.2% |
| 6254 | \| PP_Compare_rav10.olbt_s2 \| \| --- \| | 58.9% |
| 5826 | PP_Compare_sin1.statav10 | 56.6% |
| 6012 | PP_Compare_diff2.swss10_1 | 55.4% |
